# Sex matters: stress perception and the relevance of resilience and perceived social support in emerging adults

**DOI:** 10.1007/s00737-020-01076-2

**Published:** 2020-10-14

**Authors:** Nursen Yalcin-Siedentopf, Theresia Pichler, Anna-Sophia Welte, Christine M. Hoertnagl, Caroline C. Klasen, Georg Kemmler, Christian M. Siedentopf, Alex Hofer

**Affiliations:** Department of Psychiatry, Psychotherapy and Psychosomatics, Medizinische Universitat Innsbruck Department Psychiatrie und Psychotherapie, Anichstrasse 35, 6020 Innsbruck, Tyrol Austria

**Keywords:** Resilience, Stress perception, Social support perception, Sex, Emerging adulthood

## Abstract

The emerging adulthood represents a vulnerable and critical turning point for the beginning of mental illnesses and is therefore of particular interest for the study of risk and resilience. The present survey investigated the impact of sex on the associations between resilience and the perception of social support and stress in students. The Resilience Scale was used to assess resilience. Stress perception and social support perception were measured using the Perceived Stress Scale and the Social Support Questionnaire FSozU k-22, respectively. Between the ages of 18 and 30, 503 subjects (59.6% female) were included into the study. We detected a significant effect of sex with markedly lower resilience and a more pronounced perception of stress and social support among females. Significant correlations between resilience, stress perception, and social support perception were found in both sexes with women showing a stronger interrelationship between stress perception and both resilience and social support perception. Mediation analysis revealed that the relationship between the perception of social support and stress was fully mediated by resilience among men and partly mediated by resilience among women. Of note, the mediation of resilience on the interrelationship between the perception of social support and stress was much stronger in women than in men. These findings suggest that sex-specific, customized interventions focusing on the strengthening of resilience and the claiming of social support are needed to promote mental health in emerging adults.

The transition from adolescence to adulthood, also known as emerging adulthood (EA), has been described as “a time of life when many different directions remain possible, when little about the future has been decided for certain, and when the scope of independent exploration of life’s possibilities is greater for most people than it will be at any other period of the life course” (Arnett 2000). However, EA is not only a life period of personal freedom and opportunities but also a period of heightened instability, (Arnett 2000) and increased stress levels (American College Health Association 2016) which may result in an increased vulnerability for mental illnesses (Hankin and Abramson 2001; Nelson and McNamara-Barry 2005). Accordingly, EA represents a vulnerable and critical turning point in one’s life (Masten et al. 2004) that is often associated with the onset of mental disorders (Kessler et al. 2007) such as schizophrenia, mood disorders, and substance use (Hankin and Abramson 2001; Nelson and McNamara-Barry 2005; Masten et al. 2008). More generally, the EA has been associated with elevated symptoms of anxiety, depression (National Institute of Mental Health 2011), and stress (Beiter et al. 2015). Accordingly, EA is of particular interest for the study of risk and resilience.

Resilience describes the phenomenon that some individuals remain healthy or easily recover despite adverse live events, stress, and risks, while others under comparable circumstances seem to be particularly vulnerable to disorders and illnesses (Bonanno 2004). Previous studies have shown that resilience moderates the impact of functional disabilities and various forms of physical illnesses on well-being (e.g., Jason et al. 2017) and that higher levels of resilience are associated with better psychological well-being (Zhang et al. 2018) as well as lower levels of anxiety, depression (Haddadi and Besharat 2010; Wingo et al. 2010), and obsessive–compulsive symptoms (Hjemdal et al. 2011). In addition, higher levels of resilience have been associated with a decreased risk for harmful alcohol and illicit drug use in adults with a history of childhood abuse (Wingo et al. 2014) and generally with a decreased likelihood of posttraumatic stress disorder (Wrenn et al. 2011). However, there is no accordance on the operational definition of resilience. In the current study, it was considered as a positive personality trait that moderates the negative effects of adversity (Wagnild and Young 1993).

Notably, resilient subjects have been suggested to perceive less stress (Friborg et al. 2006), because they dispose of a number of supportive factors which are protective when risk or a significant threat is present and buffer against an adverse outcome (Masten and Obradović 2006). Stress has been shown to become more prevalent among EA (Beiter et al. 2015) and is one of the most impactful psychological phenomena in regard to its consequences for mental and physical health (Howland et al. 2017). In emerging adults, higher levels of stress have been shown to increase the levels of depressive and anxiety symptoms (Cano et al. 2016; Polanco-Roman et al. 2019). As a supportive factor, the meaning of social support for resilience and mental health has consistently been emphasized (Cai et al. 2017; Cohen and Wills 1985; Dos Reis et al. 2017; Juen et al. 2013; Sanders et al. 2017). Hjemdal and coworkers, for example, reported also on an association between social support and high resilience as well as low levels of depression, anxiety, and obsessive–compulsive symptoms in adolescents (Hjemdal et al. 2011). Furthermore, social support reduced the negative effects of stress (Lee and Dik 2017) and is considered as one of the most important external resources (DeLongis and Holtzman 2005), especially in EA (Gooding et al. 2012).

Resilience may be lower in female compared to male emerging adults (Lee et al. 2020). Hypothetically, these sex differences can be explained by men being more action-oriented and assertive and that they may be more likely to engage in problem-focused coping (Tamres et al. 2002), whereas women tend to ruminate when distressed and may dispose of a lower sense of mastery in their lives (Nolen-Hoeksema et al. 1999). However, females may have an advantage in regard to an improvement of resilience during this life period (Masten and Tellegen 2012). This may partly be explained by the fact that female emerging adults seek for more social support and may therefore be affected by stress to a lesser extent than males (Alcántara et al. 2015; Araújo and Borrell 2006). On the other hand, stress-induced health problems have been shown to be more frequent among female emerging adults (Zausinger et al. 2015), and a study by Zhang and coworkers found the association between social support perception and resilience to be comparable between the two sexes (Zhang et al. 2018). In order to expand on previous research, the current study therefore aimed to investigate sex-specific associations between resilience and the perception of social support and stress in emerging adults. We hypothesized that male emerging adults would show a higher degree of resilience compared to females, while women would perceive more social support and more stress than men. We further hypothesized that resilience would mediate the association between the perception of social support and stress. Lastly, we hypothesized that the association between resilience and stress perception on one hand and the association between the perception of social support and stress on the other would be stronger in females than in men and that the association between resilience and stress perception would be moderated by sex.

## Materials and methods

### Participants and procedure

Students from local universities between the ages of 18 and 30 years were recruited via the campus networks. Healthy volunteers without a history of mental health disorders or psychopharmacological treatment were included into a cross-sectional online survey. They were native German speakers and signed informed consent forms in accordance with the local ethics committee.

### Measures

The German version (Franke 2000) of the self-reported 53-item Brief Symptom Inventory (BSI) (Derogatis 1993) was used to screen for the subjective perception of global psychological distress. The BSI is a Likert-type scale, and the items are scored from 0 (not at all) to 4 (extremely). The Global Severity Index (GSI) serves as an indicator for perceived global psychological distress. The calculations were carried out with the *T* value of the GSI (GSI_T). GSI_T scores ≥ 63 are considered as clinically relevant psychological distress.

The perception of stress was measured by using of the 14-item Perceived Stress Scale (PSS-14) (Cohen et al. 1983). Items are rated on a 5-point Likert scale (0, never; 4, very often) (range, 0–56) with higher scores indicating higher levels of perceived stress. Cronbach’s alpha ranges between *α* = 0.84 and *α* = 0.86.

Resilience was assessed with the German version (Schuhmacher et al. 2005) of the 25-item Resilience Scale (RS) (Wagnild and Young 1993). Since the 2-factor structure could not be identified in the German version (Schuhmacher et al. 2005), we considered only the total score. The RS is a Likert-type scale, and items are scored on a 7-point scale from 1 (disagree) to 7 (agree) with total scores varying from 25 to 175. Higher scores indicate higher resilience. Cronbach’s alpha ranges between *α* = 0.82 and *α* = 0.95.

Social support perception was assessed with the 22-item short form of the Fragebogen zur sozialen Unterstützung (FSozU K-22) (Fydrich et al. 2007). This highly reliable (Cronbach’s *α* = 0.91) and valid questionnaire encompasses the following areas: emotional support (10 items), practical support (5 items), social integration (7 items), satisfaction with social support (2 items), and availability of a confidential person (2 items) (double assignment of four items). Each item is answered on a 5-point Likert scale ranging from 1 (agree) to 5 (disagree). A mean item score was calculated to evaluate overall social support such that a higher score indicates a higher perception of social support.

### Statistical methods

For statistical analysis, we used the statistical package SPSS, version 23. Comparisons of male and female participants regarding resilience and the perception of social support and stress were performed by means of the *t* test for independent samples. Sex-specific associations between these variables were investigated by correlation analysis using Pearson’s correlation coefficient. Comparison of correlation coefficients between male and females participants was done by means of Fisher’s r-to-z transformation.

The main part of the analysis consisted of mediation and moderation modeling. For model fitting and parameter estimation, we applied the PROCESS macro developed by Hayes (Hayes 2013). In a first step, we used simple mediation analysis (Hayes’ model 4) to investigate the supposed mediation of resilience (mediator M) on the relationship between social support perception (independent variable X) and stress perception (dependent variable Y). As the mediation effect may differ between the two sexes, this analysis was performed separately for men and women. To combine the results for both sexes, we used moderated mediation analysis as the findings of the separate analyses suggested differences in the strength of the mediation for men and women. Two different models of those proposed by Hayes were tested. In model 14 (second stage moderation model), social support perception was entered as the independent variable (*X*), stress perception as the dependent variable (*Y*), resilience as the mediator between these two variables (*M*), and sex as the moderator between resilience and stress perception (*W*). Similarly, we entered sex as a moderator between the perception of social support (*X*) and stress perception (*Y*) and between resilience (*M*) and stress perception (*Y*) in model 15 (direct effect and second stage moderation model). The preconditions for these analyses were verified. Significance was confirmed by the Sobel *z* test and bootstrapping with 10,000 bootstrap samples. All continuous variables were z-standardized prior to the mediation analyses. In addition to these analyses, we performed a second run of all mediation and moderation analyses, adding those socio-demographics to the model in which male and female participants differed significantly.

## Results

### Sample characteristics

As presented in Table 1, 503 students with a mean age of 22.7 ± 2.6 years were included into the study. There were 59.6% (*N* = 300) female. The sample had an average GSI T score of 48.1 ± 9.6. The majority of participants studied one of the following subjects: medicine, psychology, educational science, or biology. Males were significantly older than females (23.3 ± 2.8 years vs. 22.3 ± 2.4 years, *t* = 4.32, d.f. = 501, *p* < 0.001). Moreover, there were significant gender differences regarding the field of study (chi-square = 30.6, d.f. = 4, *p* < 0.001); females more frequently studied psychology (36.0% vs. 20.2% in males), while males more often chose a field of study subsumed under “other subjects”, e.g., technical subjects or sciences (25.8% vs. 10.3% in females). No other significant sex differences were found for any of the socio-demographic variables assessed, in particular partnership and living situation. Age did not show a significant relationship with any of the psychological scales used.

**Table 1:** Socio-demographic data

Variable	Category	*N (%) or mean ± SD*
Age	Years	22.7 *± 2.6*
Gender	Female	300 (59.6)
	Male	203 (40.4)
Field of study	Medicine	181 (36.0)
	Psychology	149 (29.6)
	Educational Science	23 (4.6)
	Biology	16 (3.2)
	Biotechnology	15 (3.0)
	Others*	105 (21.4)
	Missing	14 (2.8)
Partnership	In partnership	247 (49.1)
	Single	256 (50.9)

*N* = 503; others* less than ten persons (2%) per group

### Impact of sex on stress perception, resilience, and social support perception

As presented in Table 2, males showed a significantly higher degree of resilience than females, whereas female respondents achieved significantly higher scores in the perception of stress and social support.

**Table 2:** Sex differences in stress perception, resilience, and social support perception

Scale	Sex	Mean	SD	p	*t*	df	*d*
Stress perception (PSS-14)	Female	22.27	7.78	< 0.001	− 3.949	479.5	0.36
	Male	19.74	6.48				
Resilience (RS-25)	Female	136.71	16.30	< 0.001	4.550	500	0.41
	Male	143.36	15.78				
Social support perception	Female	97.47	9.30	0.038	− 2.080	500	0.19
(FSozU K-22)	Male	95.69	9.70				

*N* = 503 (females, *N* = 300; males, *N* = 203)

Abbreviations: *RS-25* Resilience Scale, *PSS-14* Perceived Stress Scale, *FSozU K-22* Fragebogen zur sozialen Unterstützung, *SD* standard deviation

d = Cohen’s effect size

### Sex-specific correlations between stress perception, resilience, and social support perception

As shown in Table 3, significant correlations between stress perception, resilience, and social support perception were found in both sexes. The largest difference between sexes was observed regarding the correlation between stress perception and resilience with women showing a stronger correlation (*r* = − .629, *p* < 0.001) than men (*r* = − .441, *p* < 0.001). This difference was highly significant (*p* = 0.004, *z* = − 2.911). Regarding the correlation between the perception of stress and social support, the sex difference missed statistical significance by a narrow margin (*p* = 0.061, *z* = − 1.868). The two sexes did not differ with regard to the correlation between social support perception and resilience (*p* = 0.562, *z* = − 0.578).

**Table 3:** Pearson correlation between age, resilience, stress perception, and social support perception, broken down by sex

	Females				Males			
Variable	1	2	3	4	1	2	3	4
1 Age	–							
2 Resilience (RS-25)	0.049	–			0.029	–		
3 Stress perception (PSS-14)	0.013	−0.629^**^	–		0.078	−0.441^**^	–	
4 Social support perception (FSozU K-22)	−0.003	0.493^**^	−0.465^**^	–	−0.036	0.532^**^	−0.321^**^	–

*N* = 503 (females, *N* = 300; males, *N* = 203), ^**^ = *p* < 0.01

Abbreviations: *RS-25* Resilience Scale, *PSS-14* Perceived Stress Scale, *FSozU K-22* Fragebogen zur sozialen Unterstützung

### Resilience as a mediator of the relation between social support perception and stress perception—separate analyses in men and women

**Fig. 1:**
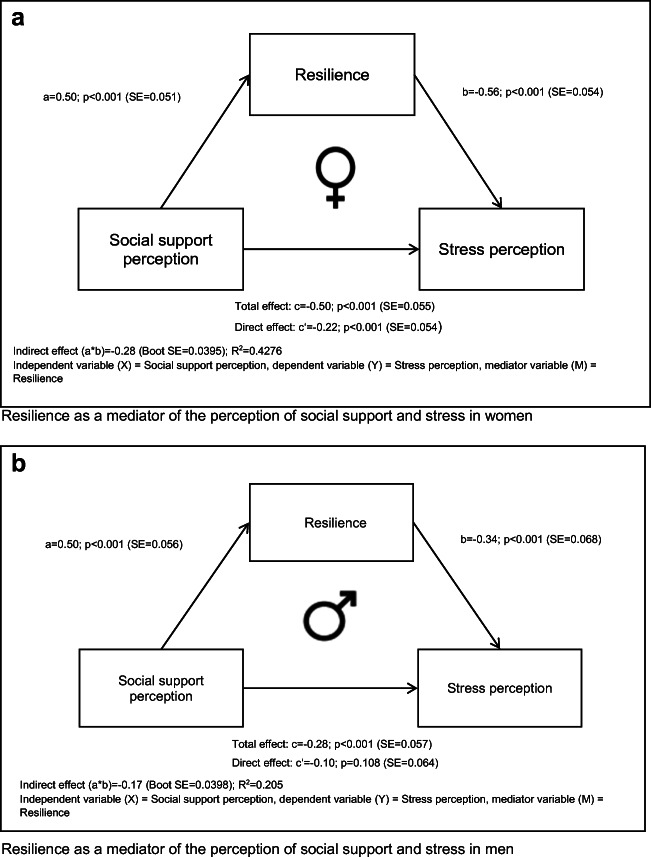
**a** Resilience as a mediator of the perception of social support and stress in women. **(b)** Resilience as a mediator of the perception of social support and stress in men

The following mediation analyses were performed separately for men and women for the sake of simplicity of modeling. Figure 1a shows the findings of the mediation analysis for women with social support perception as the independent variable (*X*), stress perception as the dependent variable (*Y*), and resilience as the mediator (*M*). The indirect effect of the mediator (a*b) was *β* = − 0.28 (CI = 0.95% [− 0.36, − 0.21], *p* < 0.001). The direct effect remained significant (*c*’ = −0.22, *p* < 0.001), indicating a partial mediation of resilience on the relationship between the perception of social support and stress among women.

Figure 1b shows the mediation for men, again with social support perception as the independent variable, stress perception as the dependent variable, and resilience as the mediator. The indirect effect of the mediator (a*b) for men showed a smaller effect than that for women with *β* = − 0.17, (CI [− 0.26, − 0.11], *p* < 0.001). In contrast to the mediation for women, the direct effect for the mediation for men did not remain significant (c’ = − 0.10, *p* = 0.108). This indicates an almost full mediation of resilience on the relation between the perception of social support and stress among men.

When adding age and field of study as covariates to the mediation model, the above results remained almost unchanged. None of the coefficients *a*, *b*, *c*, and *c’* changed by more than 0.01 in either direction.

### Combining sex-specific analyses: a moderated mediation model

To test if sex moderates both the relationship between resilience and stress perception and that between the perception of social support and stress, we used a moderated mediation model. Social support perception was again entered as the independent variable, stress perception as the dependent variable, and resilience as mediator. Sex was entered as the moderator of the relation between resilience and stress perception and between the perception of social support and stress. We found a significant interaction between resilience and sex (*β* = − 0.22, *p* = 0.013), while no significant effect of the interaction between social support perception and sex was observed (*β* = − 0.12, *p* = 0.169).

**Fig. 2:**
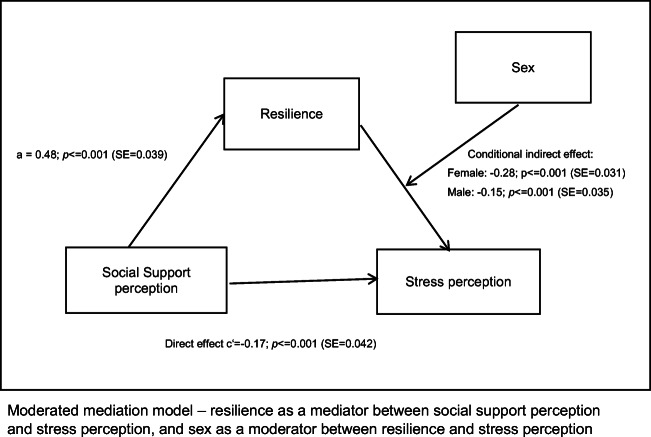
Moderated mediation model—resilience as a mediator between social support perception and stress perception, and sex as a moderator between resilience and stress perception

To account for this finding, we fitted a more parsimonious model including sex as a moderator between resilience and stress perception (*W*), but not as a moderator between perception of social support and stress (see Table 4 and Fig. 2). In line with the sex-specific results, there was a strong effect of social support perception on resilience (*β* = 0.48, *p* < 0.001). The direct effect of the perception of social support on stress perception was *β* = − 0.17 (*p* < 0.001). In this model, resilience is a partial mediator of the relation between the perception of social support and stress. The size of the indirect effect of the mediation through resilience was dependent on the moderator sex: we found smaller effects in men (*β* = − 0.15, *p* < 0.001) than in women (*β* = − 0.28, *p* < 0.001). These results imply that the mediation of resilience on the effect of social support perception on stress perception was much stronger in women than in men. The corresponding bootstrap confidence interval did not include zero (− 0.217, − 0.063), indicating that the mediation is moderated.

**Table 4:** Moderated mediation: resilience as a mediator, sex as a moderator

	Predictor	Path	*β*	SE	*p*	CI
Resilience						
X → M	FSozU	a	0.48	0.04	< 0.001	[0.40, 0.56]
Stress						
X → Y	FSozU → PSS-14 (n.V.)	c	− 0.38	0.04	< 0.001	[− 0.47, − 0.31]
X → M → Y	FSozU → RS-25 → PSS-14 (n.V.)	a*b	− 0.24	0.03	< 0.001	[− 0.30, − 0.19]
Moderated mediation: stress						
X + M + V → Y	RS-25 → PSS-14	b (if V)	− 0.03	0.13	0.84	[− 0.28, 0.26]
	FSozU → PSS-14	c’	− 0.17	0.05	< 0.001	[− 0.25, − 0.09]
	Sex → PSS-14		0.21	0.08	0.007	[0.06, 0.35]
	Sex → RS-25		− 0.28	0.08	< 0.001	[− 0.43, − 0.13]
Conditional Indirect effect						
	RS-25 (female) → PSS-14		− 0.28	0.04	< 0.001	[− 0.35, − 0.22]
	RS-25 (male) → PSS-14		− 0.15	0.03	< 0.001	[− 0.21, − 0.09]

*N* = 503 (females, *N* = 300; males, *N* = 203), *R* = 0.59; *R*^2^ = 0.34; *p* < 0.0001

CI = Bootstrapping confidence interval = 95%

X = independent variable, FSozU K-22 = Fragebogen zur sozialen Unterstützung

Y = dependent variable, PSS-14 = Perceived Stress Scale

M = Mediator = RS-25 = Resilience Scale

n. V. = without the moderator variable V

V = moderator of the mediation

a*b = Indirect effect, *c* = total effect (without V), *c’* = direct effect

As above, the results remained almost unchanged when adding age and field of study as covariates to the moderated mediation model. None of the coefficients changed by more than 0.01 in either direction.

## Discussion

The current study investigated healthy emerging adults and found a stronger interrelationship between stress perception and both resilience and social support perception in women compared to men. In addition, the relationship between the perception of social support and stress was fully mediated by resilience among men and partly mediated by resilience among women. Generally, the EA represents a critical turning point in one’s life (Masten et al. 2004) and is associated with an increased vulnerability for mental illnesses (Hankin and Abramson 2001; Nelson and McNamara-Barry 2005). Accordingly, there are wide-reaching public health implications resulting from a better understanding of sex-specific differences in the associations between social support perception, resilience, and stress perception in emerging adults (Amstadter et al. 2014) and our findings are expected to provide important implications for the promotion of mental health in young people.

In line with previous studies (Peng et al. 2012; Park et al. 2015; Tamres et al. 2002; Hilbig et al. 2015; Maciejewski et al. 2001; Padkapayeva et al. 2018), female study participants perceived more stress and social support and were less resilient than males. It has to be noted, however, that in spite of significant differences in the scores obtained in the Resilience Scale both males and females indicated moderate levels of resilience (Wagnild 2009). Following previous literature, we can speculate that women are biologically more emotional and more empathic than their male counterparts (Park et al. 2015) and therefore probably also more sensitive to stress perception which, in turn, may lead them to seek more social support than male (Adamczyk 2016). These biological differences may be based on different levels of gonadal hormones. Russo et al. (2012), for example, pointed out that testosterone promotes resilience in males and that fluctuating ovarian hormones increase the prevalence for psychiatric disorders in females.

As expected, we found a positive correlation between the perception of social support and the degree of resilience. In addition, both resilience and the perception of social support were negatively associated with stress perception, which corroborates the findings of a previous study (Zhang et al. 2018). That study revealed that promoting social support (e.g., expand social networks) improves the ability to deal with negative life events and is associated with fewer stress perception and therefore with psychological well-being.

Of note, the correlation between social support perception and resilience was comparable between the sexes in our sample, whereas the correlation between stress perception and resilience was significantly stronger in women than in men. These results differ from the findings in the above-mentioned study of Zhang et al. (2018). These mixed findings may indicate socio-cultural differences between individualistic Western countries and collectivistic Asian countries (Shi et al. 2017) and underscore the relevance of taking into account different value orientations, morals, and philosophies when investigating sex differences in mental health. It remains to be seen whether specific facets of social support (e.g., instrumental support) may have different effects on resilience and whether the two sexes differ in this regard.

The association between the perception of stress and social support was substantially stronger in females in our sample; however, this difference did not reach statistical significance.

This is of particular importance, since emerging adults without social support have been shown to be more vulnerable to health problems than adults (Lee and Dik 2017), which again underscores the relevance of promoting social support and social networks especially in this age group (Mawson et al. 2015).

In line with the findings of Brailovskaia et al. (2018), resilience mediated the interrelationship between the perception of social support and stress. Notably, this is the first study investigating sex-specific differences in this regard, and we could show that in males, the relationship between the perception of social support and stress is almost fully attributable to the mediating effect of resilience. By contrast, in females the perception of social support was related to stress perception both directly and through the mediating effect of resilience. Further, the size of the mediating effect of resilience was much stronger in females than in males.

Resilience and the perception of social support have previously been shown to be more predictive for stress perception in females than in males (Padkapayeva et al. 2018; Hjemdal et al. 2011). Accordingly, females may benefit to a greater extent from social support and improved resilience to reduce stress levels (Hjemdal et al. 2011). Our findings indicate that a reduced perception of stress and thus psychological well-being can be achieved by both improving resilience and promoting social support in women, whereas in men a reduced perception of stress can only be achieved by increasing resilience. In view of the prevention of mental illnesses, our findings therefore highlight that the two sexes may need differently weighted interventions. The better understanding of buffering factors in regard of stress perception may allow the creation of sex-specific, customized therapeutic plans to prevent mental illnesses in the EA, which, in turn, may lead to a reduction in treatment costs. The results of the current study suggest that both sexes may benefit from an improvement of resilience through training programs focusing on mindfulness and/or cognitive and behavioral skills (Joyce et al. 2018). In addition, the consolidation of personality traits like optimism, assertiveness, extraversion, flexibility, adaptability, and improvisation/innovation may be helpful in this context. On the other hand, our findings indicate that fostering social support to prevent stress perception may be of particular importance for women’s mental health. Accordingly, next to the training of resilience tailored therapeutic interventions for women should focus on the fulfillment of structural (e.g., frequency of social interactions), functional (i.e., meeting emotional or instrumental needs), emotional, instrumental/material, and informational/cognitive social needs (Southwick et al. 2016).

Despite the implications of our findings, the current study also has some limitations. First, causal relationships cannot be deduced from the findings of this study due to its cross-sectional design. Secondly, we exclusively investigated students, and selecting a sample in this way clearly limits the generalizability of the obtained results. A third limitation is the fact that most study participants were students of a health-related subject (medicine, psychology, and biology). We cannot rule out that this population might be more aware of health issues than students of other subjects. In addition, it should be considered that women experience a variety of psychological changes as well as different sensitivity for social information throughout their menstrual cycle (Lobmaier et al. 2019). This issue has not been taken into account in our study. Lastly, there are a variety of other factors influencing resilience and stress perception, which have not been taken into account in our study. Notwithstanding these limitations, this study substantially extends the insight into sex-specific differences in emerging adults. They should be taken into account when developing interventions to promote mental health in this age group.
